# Thymectomy for Myasthenia Gravis
*Abstract of an address to the Bristol Medico-Chirurgical Society on Wednesday, 13th April, 1949.


**Published:** 1949-10

**Authors:** Geoffrey Keynes

**Affiliations:** Emeritus Surgeon, St. Bartholomew's Hospital


### THYMECTOMY FOR MYASTHENIA GRAVIS*

BY

Geoffrey Keynes, M.D., F.R.C.S.

*Emeritus Surgeon, St. Bartholomew's Hospital.*

---

The thymus gland in the elderly, emaciated, dissecting-room subject is inconspicuous and, indeed, often difficult to make out. In younger subjects, who have not died after a wasting disease, it is quite a conspicuous object, relatively greatest at birth, and absolutely at puberty. It is a flattish, bilobed, glandular mass lying in the anterior mediastinum and extending from the isthmus of the thyroid gland to the pericardium. It is composed mainly of lymphoid tissue in which are embedded the epithelial Hassall's corpuscles. Its normal function is still unknown. It was formerly believed to be enlarged in the condition known as *status lymphaticus*, but this is no longer regarded as a clinical entity.

The clue to the association between the thymus gland and myasthenia gravis was provided by the observation that this disease was sometimes associated with the presence of a thymic tumour. The first successful removal of a tumour of this kind was done by Blalock of Baltimore in 1936. He took the next logical step of removing the apparently normal thymus gland in 1941, and obtained improvement in several myasthenic patients.

Myasthenia gravis is a strange disease concerning which there is still much to be learnt. The main symptom is a progressive muscular weakness, the distribution of which is very erratic. Commonly it is first noticed as diplopia, owing to weakness of the ocular muscles, so that many patients are first diagnosed in eye departments. The muscles of the face, the pharynx, the trunk and the limbs may also be affected, and, most serious of all, the respiratory muscles. The disease is remarkable for its remissions, which occasionally last for months or even years. They are scarcely ever permanent. Experienced physicians had never seen the disease in young children, but recently typical examples have been recognized in infants of two or under.

The theory of causation is connected with the action of acetylcholine at the neuro-muscular junctions. No treatment was known until the discovery by Remen[1] in 1932 of the compound known as

* Abstract of an address to the Bristol Medico-Chirurgical Society on Wednesday, 13th April, 1949.

prostigmin (now called neostigmin), and its use was introduced into this country in 1934 by Dr. Mary Walker.[2] The effect of an injection of prostigmin is apparent in a few moments ; the patient's whole appearance changes, the expressionless face becoming lively, and a patient almost moribund from respiratory failure may quickly become normal. The treatment has to be carried on by day and night for an indefinite period, some patients receiving thousands of injections, or tablets by the mouth. But prostigmin will never cure a patient, and most sufferers eventually succumb to the disease.

Some of the first operations on the thymus were done in 1910 by Victor Veau[3] for the condition known as thymic asthma. Veau made an incision at the root of the neck and pulled up a portion of the thymus, but this operation was afterwards abandoned.

Blalock's[4] approach was by splitting the sternum down the centre, and so opening the mediastinum. The speaker's first operation by this method was done in February, 1942,[5] the patient being a woman of thirty-two who was almost moribund from severe myasthenia. She made a good recovery, and for many years has been a land worker, able to do ten hour's work a day. Some 150 operations have now been done during the last seven years.

The steps of the operation (illustrated by a film in colour) are as follows. A transverse incision 3 in. long is made above the suprasternal notch, and a vertical incision is carried down from the centre of this over the sternum to the level of the fourth rib. The third intercostal space is opened on each side, and a blunt instrument is introduced into the mediastinum to meet a finger entering from above. The pleura is then pushed away on either side. The sternum is then divided transversely with a Lebsche chisel at the level of the third interspace. Finally the sternum is divided in the centre down to this level with a Sauerbruch's sternum-splitter. Oozing of blood from the cut edges of the sternum is arrested with Horsley's wax. A self-retaining retractor is inserted to expose the mediastinum for a width of three inches. The pleura is now carefully separated without injury from the surface of the thymus gland by blunt dissection. Blood-vessels entering the lateral borders are divided and ligatured. The upper cornua are separated from the thyroid isthmus, and the gland is turned downwards, exposing the large thymic vein which drains into the left innominate vein. This is divided and ligatured. The gland is then separated from the aorta and finally from the pericardium, to which it is firmly attached.

The retractor is then removed, and the two halves of the sternum are approximated by the anaesthetist, who lifts the patient's shoulders forward. The periosteum of the sternum is securely sutured with strong catgut, and the superficial layers carefully united over it. The mediastinum is not drained. Healing takes place rapidly. The patient may at first feel the edges of the sternum rubbing together with movement or coughing, but is usually able to get up out of bed within fourteen days. Since the main risk of the operation is in respiratory failure the dressing is confined to the actual area of operation and is secured by Elastoplast. No strapping or bandages are carried round the chest.

Improvement in the symptoms of the myasthenia may be noticed within a few days of the operation, but the full effect may not be obtained for weeks or months, during which the dose of prostigmin is gradually reduced.

THYMECTOMY : RESULTS IN EIGHTY-FOUR PATIENTS

| | Patients | Per cent. |
|---|---|---|
| Quite well : no prostigmin .. .. .. | 26 | 31 |
| Almost well : some prostigmin.. .. .. | 27 | 32 |
| Improved .. .. .. .. .. .. | 22 | 26 |
| No improvement .. .. .. .. .. | 9 | 11 |

There were eighty-four patients available for assessment, those who had tumours and those who died after operation being excluded, The present operative mortality was four in 100 consecutive operations. The mortality was much influenced by co-existing disease. The ultimate effect of the operation was influenced by the age of the patients and the length of the history, particularly the latter. The best results were obtained in the younger patients with the shorter histories.

Thymic tumours were found to be present in 10 per cent. of the patients. These tumours, apparently epithelial in origin, profoundly influenced the prognosis, the result of primary removal being extremely bad. They are, therefore, diagnosed, if possible, by X-rays, treated first with deep X-ray therapy, and then removed if it is feasible.

The histology of the thymus gland in myasthenic subjects is abnormal, large germinal centres being a conspicuous feature ; these are not seen in other people and their meaning is unknown.

### REFERENCES

[1] Remen, L. (1932), *Dtsch. Z. Nervenheuk*, **128**, 66.

[2] Walker, M. D. (1934,) *Lancet*, **1**, 1200.

[3] Veau, V. (1910), *Assoc. franç. de Pediatrie.*

[4] Blalock, A. (1941), *J. Amer. med. Ass.*, **117**, 1529.

[5] Keynes, G. (1946), *Brit. Journ. Surg.*, **33**, 13.

